# Association between long-term orthokeratology responses and corneal biomechanics

**DOI:** 10.1038/s41598-019-49041-z

**Published:** 2019-08-29

**Authors:** Andrew K. C. Lam, Ying Hon, Stanley Y. Y. Leung, Lu Shu-Ho, Jones Chong, David C. C. Lam

**Affiliations:** 1 Centre for Myopia Research, School of Optometry, The Hong Kong Polytechnic University, Hong Kong, China; 2 Department of Mechanical and Aerospace Engineering, The Hong Kong University of Science and Technology, Hong Kong, China

**Keywords:** Epidemiology, Outcomes research

## Abstract

Myopia is very prevalent worldwide, especially among Asian populations. Orthokeratology is a proven intervention to reduce myopia progression. The current study investigated association between baseline corneal biomechanics and orthokeratology responses, and changes of corneal biomechanics from long-term orthokeratology. We fitted 59 adult subjects having myopia between −4.00D to −5.00D with overnight orthokeratology. Corneal biomechanics was measured through dynamic bidirectional corneal applanation (in terms of corneal hysteresis, CH and corneal resistance factor, CRF) and corneal indentation (in terms of corneal stiffness, S and tangent modulus, E). Subjects with poor orthokeratology responses had lower E (mean 0.474 MPa) than subjects with good orthokeratology responses (mean 0.536 MPa). Successful orthokeratology for 6 months resulted in reducing CH (reduced by 5.8%) and CRF (reduced by 8.7%). Corneal stiffness was stable, but E showed an increasing trend. Among subjects with successful orthokeratology, a higher baseline S resulted in greater myopia reduction (Pearson correlation coefficient, r = 0.381, p = 0.02).

## Introduction

The increase in myopia has reached epidemic proportions in Asia. Holden *et al*.^1^ estimated that by 2050, more than 5.6 billion people worldwide would be affected by myopia. Recent meta-analysis reports have confirmed that orthokeratology is an effective intervention to slow the progression of myopia^2–5^. In orthokeratology, myopia reduction occurs through alteration of the anterior corneal shape^6,7^, whereas myopia control has been hypothesized to be due to myopic defocus at the peripheral retina following changes in the anterior corneal shape^8–10^. The retina is capable of responding to different types of optical defocus to drive eye growth^11,12^.

Altering the physical dimensions of the cornea may affect corneal biomechanics. After the launch of the Ocular Response Analyzer (ORA; Reichert Inc., USA) over a decade ago to clinically measure corneal biomechanics, numerous studies have confirmed changes in these measurements after orthokeratology^13–16^. Corneal hysteresis (CH), which is a measure of the viscoelastic properties of the cornea, is derived from the inward and outward intraocular pressures measured using the ORA. The corneal resistance factor (CRF) indicates overall corneal resistance. CH and/or CRF have been reported to be reduced after orthokeratology. However, few studies have investigated the association between orthokeratology responses and baseline corneal biomechanics. For example, Gonzalez-Meijome *et al*.^16^ reported that eyes with lower CH had relatively faster responses following orthokeratology, but their study involved lens wearing for only 3 hours.

Corneal indentation is an alternative method for measuring corneal biomechanics^17,18^. The corneal tangent modulus can be measured at both the central and peripheral corneas^19,20^ and is a stable parameter throughout the course of a day^21^. Our group previously studied variation in the tangent modulus after short-term orthokeratology^22^. Although short-term overnight orthokeratology did not change the tangent modulus, a higher baseline tangent modulus had greater corneal flattening along the flattest meridian. The current study incorporated both the ORA and corneal indentation to investigate the association between baseline corneal biomechanics and long-term orthokeratology responses and changes in corneal biomechanics following long-term orthokeratology. This study had two objectives: to monitor changes in corneal biomechanics from long-term successful orthokeratology and to determine which baseline parameters in corneal biomechanics were significantly associated with successful orthokeratology.

## Results

Fifty-nine subjects were eligible and fitted with orthokeratology lenses, of whom 37 completed the 6-month wearing period. These subjects included 7 males and 30 females with ages ranging from 18 to 30 years. Table 1 summarizes the reasons the 22 subjects dropped out of the study. Among these 22 subjects, 14 had poor myopia reduction from orthokeratology with a significant amount of residual refractive errors. Among the remaining 8 subjects, seven withdrew from the study within the first week of orthokeratology. Lens fitting was optimal, and we attempted to modify the lenses. Unfortunately, these 7 subjects did not wait for new lenses to arrive from overseas and discontinued the study. One subject had significant corneal staining at the 3-month follow-up visit. We advised the subject to stop wearing the lenses until complete corneal health recovery, but she decided to discontinue this study. A comparison of the baseline ocular parameters of the 37 subjects (37 eyes) who completed the study and the 14 subjects (14 eyes, randomly selected) who dropped out due to lack of an orthokeratology response or significant residual refractive error revealed that these two groups shared similar CH, CRF, and asphericity along the steepest (Steep-Q) and flattest meridians (Flat-Q) (Table 2). The eyes of the subjects who dropped out had significantly flatter keratometry results along the steepest and flattest meridians; in addition, they had similar corneal stiffness, but the normalized corneal tangent modulus was significantly lower.

**Table 1:** Summary of dropout cases.

Main reason of dropout	Number of cases
Non-responsive	2
Significant residual refractive error	12 (residual sphere was −1.90D ± 0.76D in 24 eyes)
Poor lens centration	2
Significant corneal staining	3
Lens discomfort	1
Personal reason	2

**Table 2:** Mean ± standard deviation, or median (interquartile range) of ocular parameters in the completed and dropout groups.

Parameters	Completed subjects (37 eyes from 37 subjects)	Dropout subjects (14 eyes from 14 subjects)	Comparison between two groups
Steep-K (D)	44.03 ± 1.74	43.15 ± 1.14	**t = −2.118, p = 0.041**
Flat-K (D)	42.91 ± 1.58	42.02 ± 1.13	**t = −2.227, p = 0.033**
Steep-Q	−0.184 ± 0.148	−0.115 ± 0.133	t = 1.602, p = 0.121
Flat-Q	−0.341 ± 0.098	−0.323 ± 0.106	t = 0.554, p = 0.585
CH (mmHg)	10.40 (1.50)	10.78 (1.49)	p = 0.441*
CRF (mmHg)	9.95 ± 1.30	10.31 ± 1.26	t = 0.919, p = 0.367
S (N/mm)	0.061 ± 0.007	0.058 ± 0.008	t = −0.947, p = 0.353
E_N_ (MPa)	0.536 ± 0.118	0.474 ± 0.085	**t = −3.073, p = 0.046**

Significant difference was **bold**.

Steep-K: keratometry along the steepest meridian; Flat-K: keratometry along the flattest meridian; Steep-Q: asphericity along the steepest meridian; Flat-Q: asphericity along the flattest meridian; CH: corneal hysteresis; CRF: corneal resistance factor; S: corneal stiffness; E_N_: normalized corneal tangent modulus.

Comparisons using unpaired t-tests, except CH using Mann-Whitney test*.

Table 3 shows variations in different ocular parameters throughout the study period. The refractive sphere declined rapidly within the first month and continued to reduce thereafter, albeit more slowly (RMANOVA: F = 403.4, p < 0.001). Refractive astigmatism was relatively stable initially but increased slightly at the first month and at the end of the study (RMANOVA: F = 3.910, p = 0.002). Compared with that of the baseline, a mean increase in astigmatism of 0.24 ± 0.36D was observed. The spherical equivalent (SEQ, refractive sphere plus half refractive astigmatism) was also significantly reduced throughout the study period (RMANOVA: F = 380.2, p < 0.001). The subjects achieved good and stable uncorrected visual acuity after wearing the lenses during the first week (Friedman: p < 0.001). Both the steepest and flattest corneal curvatures flattened significantly after one night and one week of orthokeratology, respectively. CCT had a small thinning effect and stabilized one week after orthokeratology (Friedman: p < 0.001).

**Table 3:** Ocular parameters throughout the study period. Results are expressed in mean ± standard deviation, or median (interquartile range).

Ocular parameter	Baseline	1 night	1 week	1 month	3 months	6 months	Statistics
Sphere (D)	−4.44 ± 0.35	−2.66 ± 0.76*	−0.87 ± 0.67*	−0.32 ± 0.60*	−0.24 ± 0.65*	0.11 ± 0.57*	**RMANOVA** **F = 403.4, p < 0.001**
Astigmatism (D)	−0.50 ± 0.33	−0.62 ± 0.38	−0.55 ± 0.36	−0.70 ± 0.45*	−0.66 ± 0.36	−0.74 ± 0.34*	**RMANOVA** **F = 3.910, p = 0.002**
SEQ (D)	−4.69 ± 0.32	−2.97 ± 0.81*	−1.15 ± 0.76*	−0.67 ± 0.68*	−0.56 ± 0.65*	−0.26 ± 0.64*	**RMANOVA** **F = 380.2, p < 0.001**
BCVA (logMAR)	−0.10 (0.10)	−0.10 (0.10)	−0.10 (0.10)	−0.10 (0.10)	−0.10 (0.10)	−0.08 (0.10)	Friedman, p = 0.110
UCVA (logMAR)	1.02 (0.16)	0.70 (0.36)	0.10 (0.14)*	0.02 (0.18)*	0.01 (0.14)*	0.00 (0.16)*	**Friedman**, **p < 0.001**
Steep-K (D)	44.03 ± 1.74	43.40 ± 1.68*	42.44 ± 1.68*	42.05 ± 1.67*	42.08 ± 1.66*	42.09 ± 1.66*	**RMANOVA** **F = 140.0, p < 0.001**
Flat-K (D)	42.87 (2.23)	42.17 (1.99)	41.48 (2.07)*	40.95 (2.23)*	41.14 (2.17)*	40.92 (1.93)*	**Friedman**, **p < 0.001**
CCT (µm)	545.3 (42.7)	546.0 (43.3)	537.7 (43.7)*	535.0 (49.0)*	538.3 (47.7)*	538.0 (44.3)*	**Friedman**, **p < 0.001**
CH (mmHg)	10.53 ± 1.19	10.22 ± 1.17*	10.00 ± 1.26*	9.99 ± 1.12*	9.91 ± 1.22*	9.92 ± 1.25*	**RMANOVA** **F = 9.320, p < 0.001**
CRF (mmHg)	9.90 (1.80)	9.67 (1.77)	9.30 (1.70)*	9.10 (1.87)*	8.97 (1.60)*	8.90 (1.47)*	**Friedman**, **p < 0.001**
S (N/mm)	0.060 (0.008)	0.060 (0.010)	0.059 (0.007)	0.057 (0.006)	0.059 (0.007)	0.057 (0.005)	Friedman, p = 0.182
E_N_ (MPa)	0.536 ± 0.118	0.539 ± 0.129	0.562 ± 0.093	0.578 ± 0.116*	0.579 ± 0.097*	0.571 ± 0.106	**RMANOVA** **F = 3.263**, **p = 0.008**

Significant difference was **bold**.

RMANOVA: repeated measures analysis of variance.

*post-hoc test (Dunnett’s method) showing significant difference with baseline.

SEQ: spherical equivalent; BCVA: best corrected visual acuity; UCVA: uncorrected visual acuity; Steep-K: keratometry along the steepest meridian; Flat-K: keratometry along the flattest meridian; CCT: central corneal thickness; CH: corneal hysteresis; CRF: corneal resistance factor; S: corneal stiffness; E_N_: normalized corneal tangent modulus.

Both CH and CRF decreased throughout the study. Compared with those at the baseline, the CRF at 6 months was decreased by 8.7% (−0.87 ± 0.76 mmHg), and CH was decreased by 5.8% (−0.61 ± 0.76 mmHg). Corneal stiffness did not change significantly, but the normalized tangent modulus showed an increasing trend (Table 3), this measure was significantly higher than the baseline after one month and three months of orthokeratology.

**Figure 1:**
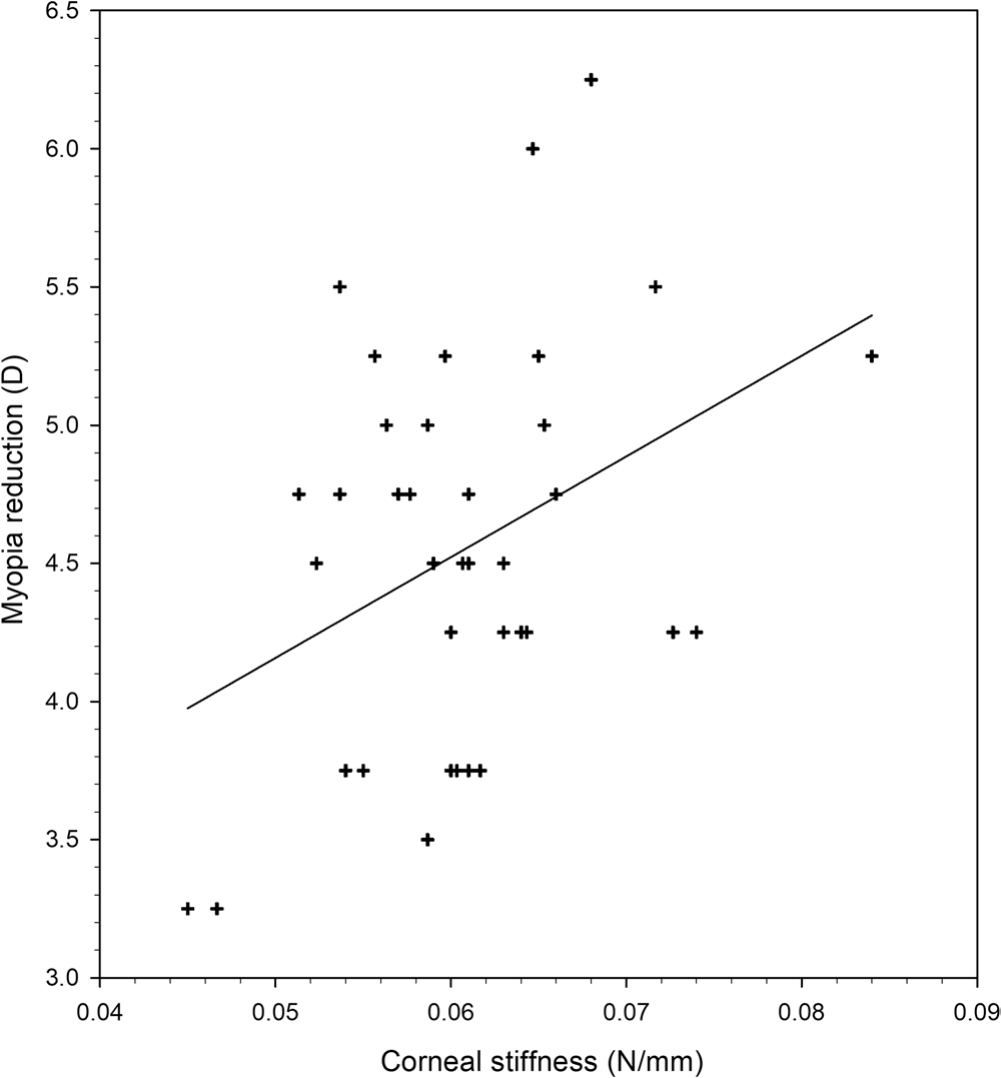
Linear regression analysis between myopia reduction at the 6th month of orthokeratology and baseline corneal stiffness. Pearson correlation coefficient (r) is 0.381, p = 0.02. Regression equation is y = 36.466x + 2.334.

Only the baseline corneal stiffness demonstrated a significant correlation with sphere reduction after 6 months of orthokeratology (r = 0.381, p = 0.020). A linear regression model revealed how the myopia reduction was related to the baseline corneal stiffness (Fig. 1). The baseline tangent modulus was positively correlated with myopia reduction, but this correlation did not reach statistical significance (r = 0.311, p = 0.061). Correlations between other ocular parameters (namely CH, CRF, steepest and flattest keratometry, steepest and flattest asphericity) and myopia reduction were even weaker (Pearson’s correlation coefficient or Spearman’s rho ranged between 0.045 and 0.188).

We calculated the post hoc power using G-power version 3.1.9.2 (Franz Faul, Universität Kiel, Germany). Based on the 5.8% decrease in CH after 6 months of orthokeratology, the current study had over 99% power to detect changes in corneal biomechanics at an alpha value of 5%.

## Discussion

Orthokeratology is an effective technique to temporarily reduce myopia^23^ and even reduce myopia progression^24–26^. This technique alters the corneal dimensions by flattening the anterior curvature^6,27^, reducing the central thickness, and increasing the mid-peripheral thickness^28^. After orthokeratology, the corneal epithelium is reduced^29,30^, but the corneal stroma thickens^31,32^. These changes in the morphological and histological properties of the cornea may alter its biomechanical properties^33^. The current study applied both a dynamic bidirectional corneal applanation technology (i.e. the ORA) and corneal indentation to monitor changes in corneal biomechanics through the 6-month orthokeratology period. The corneal stiffness was stable, whereas the tangent modulus exhibited a significant increasing trend (Table 3). Previous studies that have used the ORA have reported changes in CH or the CRF^14–16,34^.

Following initiation of orthokeratology, our subjects showed a significant reduction in the spherical component during the first month, and good uncorrected visual acuity was achieved in the first week (Table 3). These findings are consistent with those of previous studies that have used the same type of lens^35^. By contrast, the astigmatic component increased slightly. Orthokeratology usually cannot provide a significant reduction in refractive astigmatism. Even for studies using toric orthokeratology lenses, the purpose was mainly to improve lens centration rather than reducing astigmatism totally^36–38^.

We previously reported a reduction in the CRF with a relatively stable CH after one session of overnight orthokeratology^15,22^. Orthokeratology for longer periods exerts different effects on CH and the CRF. Chen *et al*.^33^ reported that both CH and the CRF declined significantly after one week of orthokeratology and that the reduction in the CRF was greater. This result was affirmed by Yeh *et al*.^13^, who monitored CH and the CRF for one month. The reduction in the CRF was greater (7.4%) than that in CH (2.8%). Moreover, Nieto-Bona *et al*.^14^ reported a similar reduction in CH and the CRF (6%) after one month of orthokeratology. Fewer studies have monitored CH and CRF throughout the course of long-term orthokeratology. One study in China found a significant initial drop in both CH and the CRF, but these parameters returned to the pretreatment levels after 3 months of orthokeratology^34^. The current study found significant reductions in both CH and the CRF throughout the 6-month orthokeratology period, with a greater reduction in the CRF. As shown in Table 3, the CH reduction plateaued after one month of orthokeratology whereas the CRF exhibited a sustained decreasing trend. Since the corneal stroma constitutes 90% of the entire corneal thickness, CH and the CRF are mainly associated with the corneal stromal thickness^39^. Supporting this hypothesis, Chen *et al*.^33^ reported significant associations of changes in CH and the CRF with changes in the stromal thickness.

Studying corneal biomechanics using the ORA has certain drawbacks because of the wide inter-subject variation in CH and the CRF^13^. Corneal indentation is an alternative corneal biomechanics measurement method *in vivo*^19^. Corneal stiffness is defined as the force required to indent a cornea to a particular depth (1 mm in our device). This biomechanical property depends on the physical properties of the cornea, including its thickness and radius. Tangent modulus is an intrinsic mechanical property of a tissue. Hon *et al*.^40^ used both the ORA and corneal indentation to compare the corneal biomechanics of low and high myopes. Although high myopes exhibited lower mean CH than low myopes, as documented in the literature, the two groups demonstrated nearly the same CH spread due to wide inter-subject variation. Furthermore, high myopes had not only a lower mean tangent modulus but also a narrow spread of the tangent modulus.

One novel finding of the current study is that long-term orthokeratology increases the tangent modulus while the corneal stiffness remains stable. This result implies that even though the geometric properties (i.e. thickness and radius) of the cornea change, some physical properties (e.g. stiffness) do not. However, orthokeratology altered the intrinsic material properties of the cornea. A thorough evaluation of the sub-corneal layers is required, because these layers have different elastic properties^41,42^. For example, Zhong *et al*.^43^ found that the basal cell and stromal keratocyte densities declined after 5 years of orthokeratology. Nieto-Bona *et al*.^44^ obtained similar findings for the basal cell and anterior stromal keratocyte densities after 6 and 12 months of orthokeratology. In addition, they reported thinning of Bowman’s membrane during the same period and speculated that imaging artefacts due to epithelial compression might affect this measurement. Among the anterior sub-corneal layers, Bowman’s membrane has the highest elastic modulus, followed by the stroma among the other anterior sub-corneal layers^41,42^. Confocal microscopy can be incorporated to measure Bowman’s membrane and the stromal thickness in future studies. Owens *et al*.^45^ compared rabbit eyes after two weeks of overnight orthokeratology with untreated eyes and did not find a significant difference in the stromal collagen fibril diameter. *In vivo* measurement of corneal collagen fibres may further enrich our understanding of corneal biomechanics^46,47^.

One objective of our study was to evaluate whether baseline corneal biomechanics had significant associations with the orthokeratology success (i.e. myopia reduction). Myopia reduction was used as an indicator of the orthokeratology success because it was more than flattening of the corneal curvatures^48–50^. Furthermore, myopes seek orthokeratology for myopia reduction. In the 59 subjects fitted with orthokeratology lenses, 14 did not have a good orthokeratology response and thus dropped out of this study. When we compared the ocular parameters between the subjects who completed the 6-month orthokeratology (37 eyes of 37 subjects) and those who dropped out (14 eyes of 14 subjects), the dropout group had a flatter baseline corneal curvature and a significantly lower tangent modulus (Table 2). In myopic orthokeratology, the anterior corneal curvature is flattened^6,27^; thus, myopia reduction will be limited if the baseline corneal curvature is flat and spherical^51,52^. The dropout group had a flatter corneal curvature, but their corneal asphericity was similar to that of the completed group. The dropout could also be related to the lenses (Menicon Z Night contact lenses) used in the current study. Santodomingo-Rubido *et al*.^50^ used the same lens type and reported that only 68% of children could achieve an optimum lens fit from the first contact lens. Moreover, 32% of their subjects required a total of 35 adjustments to obtain an optimum lens fit. Chan *et al*.^35^ reported that myopia reduction only reached 57% and 81% after wearing these lenses for one night and one week, respectively. The prolonged trial period due to many lens modifications discouraged our adult subjects from continuing the study. Our lenses had fenestrations for better corneal health that could further reduce the rate of myopia reduction^53^. Among the measured corneal biomechanics, only the tangent modulus differed between the two groups; the baseline tangent modulus of the group that completed the study was 0.536 MPa, which was comparable with that of healthy subjects^40^, Hon *et al*.^40^ reported a mean tangent modulus of 0.57 MPa in low myopes (mean SER of −1.37D) and 0.47 MPa in high myopes (mean SER of −9.07D). The mean tangent modulus of our dropout group (0.474 MPa) was low in myopes with myopia between −4.00D and −5.00D. Therefore, incorporating an evaluation of corneal biomechanics may help screen good orthokeratology responders. However, orthokeratology practitioners should refer to both corneal curvatures and biomechanics (Table 2). Vinciguerra *et al*.^54^ suggested integrating biomechanical evaluation with tomographic and topographic analyses for diagnosis of subclinical keratoconus.

We performed a correlation analysis for the group who completed the study, but except for corneal stiffness, no other baseline corneal parameter demonstrated a significant association with myopia reduction. Gonzalez-Meijome *et al*.^16^ was the first study to investigate whether orthokeratology responses were correlated with corneal biomechanics. They proposed that corneas with a low CH or CRF might respond faster to myopic orthokeratology. They used corneal curvatures as the response of interest, whereas we used myopia reduction in this study, because orthokeratology resulted in more myopia reduction than flattening of the corneal curvature^48–50^. The difference in the results could be due to different wearing conditions: our subjects wore the lenses during sleep, whereas their subjects wore the lenses for only 3 hours and under an open-eye condition^16^. Furthermore, we set stringent inclusion criteria related to age and the myopia range to account for confounding factors that could affect corneal biomechanics. We concluded that neither the baseline CH nor CRF had a significant association with myopia reduction during long-term orthokeratology.

In the current study, corneal stiffness was defined as the force required to induce unit corneal displacement. This measure is influenced by the corneal dimensions, including its curvature and thickness. Corneas with a higher baseline stiffness appear to lead to greater myopia reduction (Fig. 1). However, we should interpret this result with caution. Our data points were very scattered, and higher corneal stiffness only accounted for 14.5% (r = 0.381) of the cases with greater myopia reduction. Therefore, other factors should contribute to the higher myopia reduction in these eyes. Neither central corneal curvatures nor asphericity had significant correlations with myopia reduction. Consistent with our observation, Chan *et al*.^55^ previously reported that corneal asphericity could not predict myopia reduction after overnight orthokeratology.

Other factors could affect the performance of overnight orthokeratology. Younger patients, such as children, were more likely to have adverse effects from overnight lens wear^56^. The incorporation of fenestrations in our orthokeratology lenses could reduce lens binding^53^. Additionally, the experience of the practitioner is crucial in orthokeratology^57^. Our frequent aftercare visit schedule, which involved an experienced orthokeratology practitioner, and conduct of the study at a university clinic ensured a high standard of performance^58^.

The current study monitored corneal biomechanics in long-term orthokeratology using two technologies: dynamic bidirectional corneal applanation and corneal indentation. The strength of this study lies in its stringent inclusion criteria regarding baseline myopia and age, because corneal biomechanics differ with age and refractive groups. However, our study has several limitations. Corneal biomechanics is age-dependent^59–62^. Our subjects were all adults, although orthokeratology usually is offered to children for myopia control. Answering the question of whether our findings apply to children requires further research. Corneal responses could vary based on the orthokeratology lens design^63^. Further studies can include confocal microscopy to evaluate histological changes. We concede that no single parameter can adequately describe all corneal biomechanical properties. CH represents the viscoelastic property of the cornea^62^; thus, evaluating corneal deformation and deflection through dynamic Scheimpflug imaging is another promising technology to study corneal biomechanics *in vivo*^64,65^.

To conclude, intrinsic corneal tissue properties together with corneal curvature contribute to successful overnight orthokeratology. A cornea with a flat corneal curvature together with an unusually low tangent modulus may not respond well to myopic orthokeratology. In successful overnight orthokeratology, the baseline corneal stiffness has a weak but significant association with myopia reduction.

### Data collection

#### Subjects

In total, 275 subjects were screened for eligibility to participate in this study. The inclusion criteria were an age between 18 and 30 years, myopia between −4.00D and −5.00D in sphere power, and with-the-rule astigmatism within 1.50D. In addition, differences in refractive errors for both the sphere and astigmatic components, of the left and right eyes were required to be within 1.00D. These stringent criteria eliminated confounding factors in corneal biomechanics, such as age^61,66^ and myopia^39,40^. The exclusion criteria were long-term contact lens use or a history of ocular diseases. In addition, those with a best-corrected visual acuity of less than 0.10 logMAR in each eye measured using the Early Treatment Diabetic Retinopathy Study chart (Prevision Vision, La Salle, IL) under normal room lighting conditions were excluded. All procedures were performed in accordance with the ethical standards of the institution and the 1964 Declaration of Helsinki. Ethics clearance was obtained from the institutional review board of The Hong Kong Polytechnic University. Informed consent was obtained from all participants included in the study. This study was registered at ClinicalTrials.gov (NCT02719535, registered March 25, 2016) and in the University of Hong Kong HKU Clinical Trials Register (HKUCTR-1957, registered Feb 1, 2016).

## Methods

The following baseline data were collected: non-cycloplegic manifest refraction, ocular biometry through partial coherence interferometry (Zeiss IOLMaster; Zeiss Humphrey, Dublin, CA), corneal topography (Medmont E300, Medmont Pty Ltd., Vermont, VIC, Australia), corneal thickness through swept-source optical coherence tomography (Casia SS-1000, Tomey, Nagoya, Japan), and corneal biomechanics using an ORA. CH, CRF, and corneal-compensated intraocular pressure (IOPcc) were measured using the ORA. Three acquisitions were obtained, each with a waveform score of at least 6.0^67^. The CRF is a measure of the overall resistance of the cornea. The IOPcc is a measure of intraocular pressure (IOP) and is less affected by corneal parameters, such as the central corneal thickness (CCT)^68^. After all non-contact procedures were completed, the cornea was anaesthetized using one drop of 0.4% benoxinate. The IOP was measured through Goldmann applanation tonometry, followed by corneal indentation. The corneal stiffness and tangent modulus measurements were described in detail in the literature^19^. Briefly, first, the indenter is brought into contact with the central cornea. Then, the preload is stabilized (as confirmed by an audible sound), and the indenter is moved forwards and backwards at 12 mm/s to indent the cornea by 1 mm. Each indentation is completed in less than 0.25 seconds. Finally, the corneal stiffness is read from the indentation device. In this study, the average stiffness from three measurements was used to calculate the tangent modulus using the central corneal radius and CCT. Because the corneal tangent modulus varies with the IOP^17^, the tangent modulus was normalized to the mean IOP in normal eyes (15.5 mmHg) using the IOPcc^40^.

### Lenses used

Menicon Z Night contact lenses (NKL Contactlenzen, Netherlands) made of super-high gas permeable material (Dk: 163 × 10^−11^) were used. The back optic zone radius of the lenses ranged from 7.20 to 9.50 mm in 0.05 mm steps. The lens diameters were either 10.20 mm or 10.60 mm. Three fenestrations were located in the reverse curve 120°apart to enhance tear exchange. The lenses were fitted according to the manufacturer’s instructions using a computer programme (Easy Fit Software, Menicon Co Ltd., Nagoya, Japan). This computer-assisted lens fitting method has a very high first-fit success rate for myopic orthokeratology in low to moderate myopes^35^. Lens fitting requires ocular information, such as corneal topography and non-cycloplegic manifest refraction. The required lenses were ordered, and a trial fitting was arranged.

### Wearing schedule

After a successful trial fitting, a delivery visit was arranged. Subjects were asked to return to the University Optometry Clinic for regular follow-up per the following schedule: after the first overnight wear and after 1 week, 1 month, 3 months, and 6 months of lens wear. Each visit was completed within two hours of waking in the morning and removing the lenses. Lens parameters were modified when necessary, especially in case of poor lens centration or significant residual refractive error.

At each follow-up visit, the corneal topography, thickness, and biomechanics were measured using both the ORA and corneal indentation in addition to the subjective refraction and ocular biometry. Both the habitual uncorrected visual acuity (UCVA) and best-corrected visual acuity (BCVA) were measured.

### Statistical analysis

Data of one eye of each subject who completed the 6-month orthokeratology were analyzed. Only one eye per subject was included to avoid inter-eye correlation that could influence the analysis results. We compared the refractive sphere, refractive astigmatism, and BCVA between the two eyes at the 6-month visit. No significant difference was found between the two eyes (refractive sphere, p = 0.657; refractive astigmatism, p = 0.364; BCVA, p = 0.390). Since we used myopia reduction rather than corneal flattening to represent orthokeratology success^48–50^, the eye with the residual sphere closer to plano at the 6-month visit was selected. If the residual sphere was the same in both eyes, then the eye with less residual astigmatism was selected. For subjects with the same residual refractive errors in both eyes, the right eye was selected. Some previous studies also selected a “better” eye rather than using a random order to select an eye for data analysis^69–71^. Normality was checked using the Shapiro-Wilks test. Repeated-measure analysis of variance (RMANOVA) or the Friedman test was used to compare changes in ocular parameters throughout the study (i.e. from baseline to the end of the 6-month orthokeratology period). When a significant difference was found, a post hoc test (Dunnett’s method) was used to compare results from the different follow-up visits with the baseline data. Baseline ocular parameters that were significantly correlated (Pearson or Spearman) with myopia reduction were identified. A linear regression model was applied when a significant correlation was found. Reduction of the sphere at the 6-month visit was used as the dependent outcome response, and the various baseline ocular parameters were treated as independent predictors. All data analyses and graphical presentations were completed using SigmaPlot 13 (Systat Software, Inc.).
